# CRISPR-Cas13d Exhibits Robust Antiviral Activity Against Seneca Valley Virus

**DOI:** 10.3389/fmicb.2022.835040

**Published:** 2022-02-14

**Authors:** Yu-Yuan Zhang, Ming-Xia Sun, Yuexiao Lian, Tong-Yun Wang, Mei-Yu Jia, Chaoliang Leng, Meng Chen, Yuan-Zhe Bai, Fandan Meng, Xue-Hui Cai, Yan-Dong Tang

**Affiliations:** ^1^State Key Laboratory of Veterinary Biotechnology, Harbin Veterinary Research Institute of Chinese Academy of Agricultural Sciences, Harbin, China; ^2^Guangdong Laboratory Animals Monitoring Institute and Guangdong Key Laboratory of Laboratory Animals, Guangzhou, China; ^3^Henan Provincial Engineering and Technology Center of Animal Disease Diagnosis and Integrated Control, Nanyang Normal University, Nanyang, China

**Keywords:** CRISPR-Cas13d, CasRx, SVV, antiviral, virus

## Abstract

In recent years, Seneca Valley virus (SVV) as a newly identified pathogen of porcine vesicular disease spread quickly and has posed a potential threat to the swine industry in several countries resulting in economic losses. Considering the evolution of SVV, attention should be given to controlling SVV epidemics. So far there are no commercial vaccines or drugs available to combat SVV. Therefore, development of strategies for preventing and controlling SVV infection should be taken into account. In the current study, we evaluated whether the CRISPR-Cas13d system could be used as a powerful tool against SVV infection. Besides, selected crRNAs showed different capacity against SVV infection. Our study suggests the CRISPR-Cas13d system significantly inhibited SVV replication and exhibited potent anti-SVV activity. This knowledge may provide a novel alternative strategy to control epidemics of SVV in the future.

## Introduction

Seneca Valley virus (SVV), also known as Senecavirus A, causes vesicular disease in pigs, and it is a non-enveloped, single-stranded RNA virus that belongs to the genus Senecavirus in the family *Picornaviridae* (Hales et al., 2008). SVV was first isolated and identified as a cell culture contaminant in the United States in 2002 (Reddy et al., 2007). Initially, SVV was mainly used as a potential oncolytic virus in cancer therapy, and there was no evidence that SVV was pathogenic to farm animals (Burke, 2016). However, in recent years, SVV has been reported to cause outbreaks in many countries and is associated with porcine vesicular disease (Hause et al., 2016; Leme et al., 2016; Zhao et al., 2017; Zhu et al., 2017; Zhang et al., 2018). The virulence of SVV seems to have increased when it evolved in pigs, and some newly emerged SVV strains lead to acute death of neonatal piglets (Leme et al., 2016, 2017). Currently, there are no commercial vaccines or drugs available to combat SVV.

The CRISPR-Cas system is recognized as the “adaptive” immune system of bacteria or archaea, protecting these organisms from invasions of viral infection, plasmid transfer, and foreign DNA interference (Horvath and Barrangou, 2010; Liao et al., 2021). CRISPR-Cas9 system has been reported to inhibit DNA virus replication effectively (Tang et al., 2016, 2017, 2018). Recently, the CRISPR-Cas13d system is an RNA-guided, RNA-targeting CRISPR system that exhibits high knockdown efficiency and strong specificity compared to RNA interference (Konermann et al., 2018). CRISPR-associated RNAs (crRNAs) contain an approximately 22-nt spacer sequence that guides the Cas13d protein to target RNA for degradation. Cas13d derived from *Ruminococcus flavefaciens* XPD3002, also known as CasRx, shows the best knockdown activity in mammalian cells (Konermann et al., 2018). Recently, the CRISPR-Cas13d system has been used to combat several RNA viruses, including SARS-CoV-2, and exhibits potent antiviral activity (Abbott et al., 2020; Nguyen et al., 2020; Lin et al., 2021).

## Materials and Methods

### Cells, Virus, Reagents, and Plasmids

Swine testicle (ST) cells and human embryonic kidney (HEK293T) cells were maintained in Dulbecco’s modified Eagle medium (DMEM; Thermo Fisher, United States) supplemented with 10% fetal bovine serum as previously described (Yang et al., 2020, 2021).

The Senecavirus A strain (GD strain) was isolated and sequenced in our laboratory (unpublished data). The virus was propagated and titered in ST cells. Viral titers were determined by a 50% tissue culture infectious dose (TCID50) assay in ST cells. Viral titers were calculated using the Reed and Muench method (Reed and Muench, 1938).

The anti-HA monoclonal antibody (H9658) was purchased from Sigma. The VP2 monoclonal antibody was produced and preserved by our laboratory. The secondary antibodies used for immunofluorescence analyses were Alexa Fluor 568 goat anti-rabbit or mouse IgG (H + L; Thermo Fisher, United States). The cellular nuclei were stained with 4′,6-diamidino-2-phenylindole (DAPI, Beyotime, Beijing, China).

### CRISPR-Cas13d System

The RfxCas13d expression vector pXR001 EF1a-CasRx-2A-EGFP was obtained from Addgene (Addgene, United States). To construct CasRx-del-NSL, two individual nuclear localization signals were removed from the EF1a-CasRx-2A-EGFP plasmid. Briefly, overlapping PCR was performed, and the construct was recloned into the original vector. The four specific crRNA targeting sites were as follows: crRNA#1: TGCATTTCCATAAGAGAGAGCGC, crRNA#2: CATTTCCATAAGAGAGAGCGCTC, crRNA#3: CCAACATAGAAACAGATTGCAGC, and crRNA#4: ATCGTCAGACATTTCCACCCACT. Guide RNA was synthesized as DNA oligos, and then, each crRNA was cloned into the pXR003 CasRx crRNA cloning backbone (Addgene, United States).

### Virus Replication Inhibition Assay

HEK-293T cells were seeded in 12-well plates, and 1 μg of CasRx and 1 μg of the indicated crRNA expression vector were cotransfected into 293T cells. Twelve hours later, the cells were infected with SVV at an MOI of 0.01. The virus was collected 24 and 48 hpi. The titers of SVV were determined by the TCID50 assay. IFA was performed as described in our previous work (Zhang et al., 2021).

### Colocalization Coefficients Analysis

HEK-293T cells were seeded in 12-well plates, and 1 μg of CasRx and 1 μg of the indicated crRNA plasmid or mix pool (contain four indicated crRNA at equal ratio) were cotransfected into 293T cells. Twelve hours later, the cells were infected with SVV at an MOI of 0.36 random selected images were analyzed by ImageJ. Manders’ Colocalization Coefficients (Manders’ M relate to EGFP) was analysis.

### Cell Viability Assay

HEK293T cells were seeded in 96-well plates, and 0.05 μg of the indicated crRNA plasmid (#1, #2, #3, #4, or Mix pool) and 0.05 μg of CasRx were cotransfected into 293T cells. Viability of cells was determined using the Cell Counting Kit-8 (Dojindo, catalog number CK04). Ten microliter of the cck-8 solution was added after 24 or 48 h of incubation. Subject to incubation at 37°C for 2–4 h, the absorbance at 450 nm was measured by An Absorbance Microplate Reader (The ELx808TM).

### Statistical Analysis

Statistical significance was analyzed with one-way ANOVA and Tukey’s multiple comparison test or the independent-samples Student’s *t*-test in Origin GraphPad Prism 8.0 software. All differences with value of *p* < 0.05 were considered statistically significant.

## Results and Discussion

**Figure 1:**
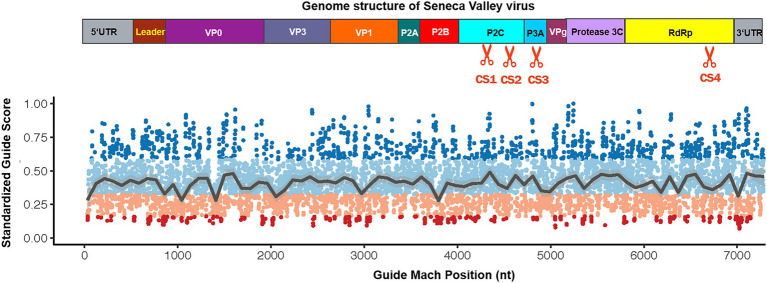
Schematic diagram depicting the guide RNA match position in the Seneca Valley virus (SVV) genome. Protein construction from the SVV genome transcript, which demonstrates the cleavage site of the most effective guide RNA in this study (**upper panel**). Depiction of the score distribution along SVV genome; high scores indicate a higher predicted knockdown efficacy (guide scores range between 0 and 1; **lower panel**).

**Figure 2:**
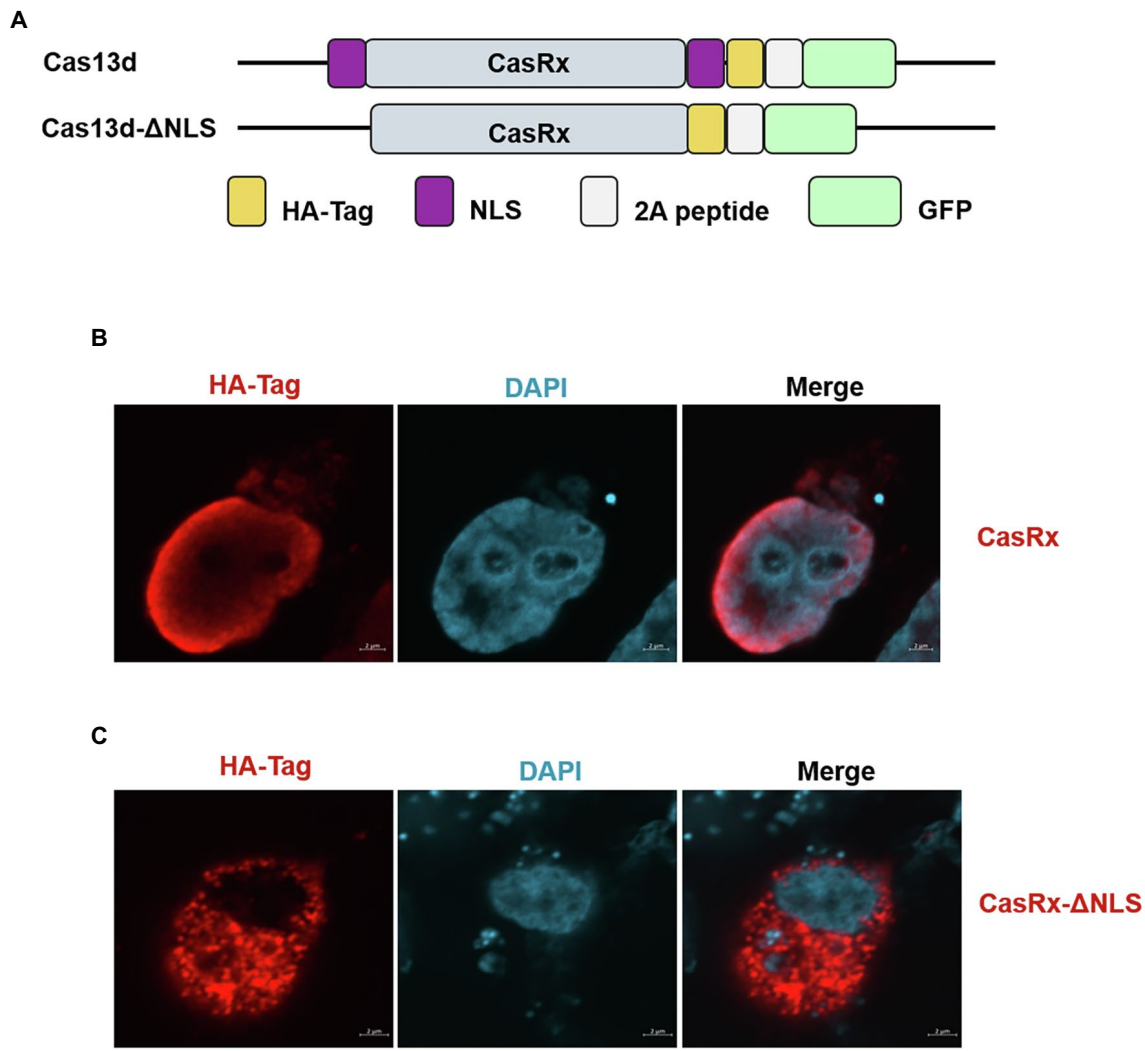
Deletion of the nuclear localization signal (NLS) regulates Cas13d subcellular localization. **(A)** Summary of the constructs used in this study. Cas13d contains two NLSs that allow Cas13d to enter the nucleus. Removal of the NLS enables Cas13d to cleave the complementary target RNA within the cytoplasm but not the nucleus. **(B)** HEK293T cells were transfected with the CasRx expression vector or **(C)** CasRx-del-NLS, and then, IFA was performed at 24 hpi; the HA tag at the C-terminus of Cas13d was stained and labeled with Alexa Fluor 568-conjugated goat antibodies. Nuclei were labeled with DAPI. Scale bar = 2 μm.

In the current study, we tested whether the CRISPR-Cas13d system could be used as an effective antiviral strategy to combat SVV. The SVV strain in this study was isolated and sequenced in our laboratory (unpublished data). The genome structure of SVV is shown in Figure 1 (upper panel). To predict the possible crRNA, we first input the whole genome sequence into the predicted software, which is available at https://gitlab.com/sanjanalab/cas13 (Wessels et al., 2020). In software screening, potential crRNA sequences were listed and ranked by their predicted standardized guide scores (Figure 1). We selected four crRNAs with high scores (two targeting P2C, one targeting P3A, and one targeting RdRp) from the list for further investigation. Because SVV mainly undergoes replication and viral RNA synthesis in the cytoplasm rather than the nucleus (Flather and Semler, 2015), CasRx had to be engineered to localize to the cytoplasm for RNA cleavage. The CasRx expression cassette for cellular mRNA knockdown is shown in Figure 2A, and we next removed the two nuclear localization signals (NLSs) from CasRx (CasRx-del-NSL). By immunofluorescence detection, we found that CasRx-del-NSL was successfully expressed and mainly located in the cytoplasm (Figure 2C). As a control, CasRx was retained in the nucleus, as expected (Figure 2B).

**Figure 3:**
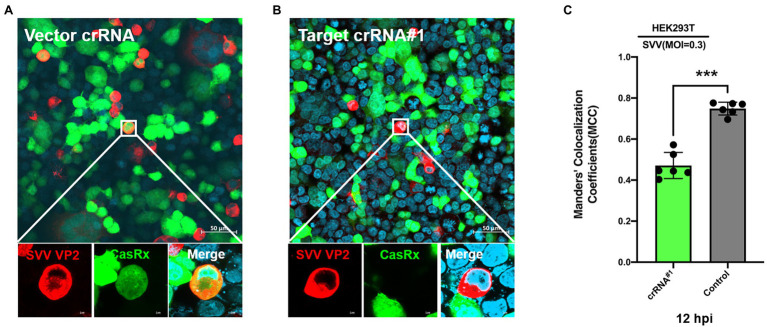
Programmable targeting of SVV RNA by Cas13d. The CasRx-del-NLS plasmid cotransfected with **(A)** control crRNA plasmid or **(B)** targeted crRNA#1 plasmid into HEK293T cells. At 24 hpi, the cells were infected with SVV (at an MOI of .3). Thirty-six hours post-infection, SVV VP2 was detected by IFA with an anti-VP2 monoclonal antibody and Alexa Fluor 568-conjugated goat antibodies. CasRx expression was indicated by EGFP, and nuclei were labeled with DAPI. **(C)** Manders’ colocalization coefficients was analyzed, six random selected images were analyzed by ImageJ. ^***^p < 0.001.

**Figure 4:**
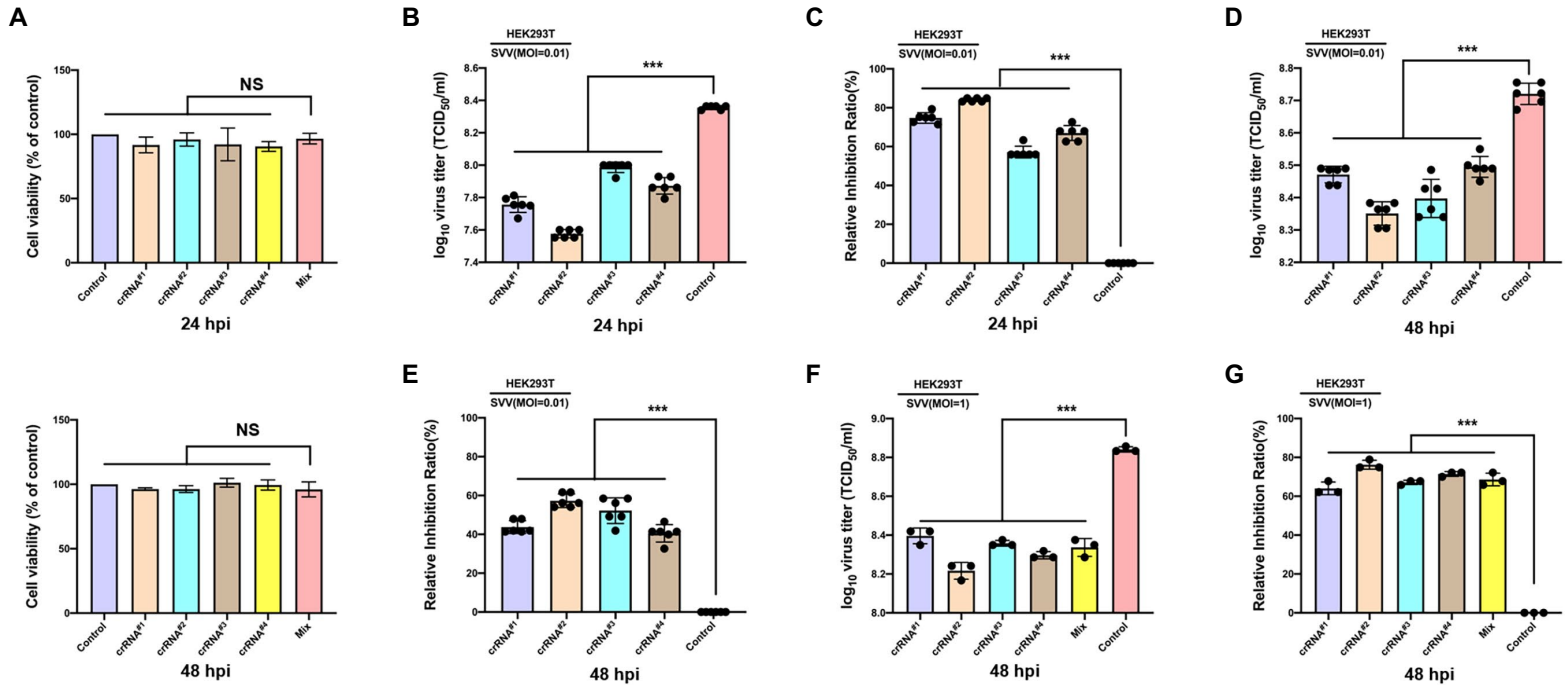
Efficient inhibition of SVV infection *via* the engineered CRISPR-Cas13d system. **(A)** HEK-293T cells were cotransfected with Cas13d and the indicated crRNAs plasmid, the cells viability at 24 hpt or 48 hpt was determined. **(B)** HEK-293T cells were cotransfected with Cas13d and the indicated crRNAs plasmid. Twenty-four hours later, the cells were infected with SVV (at an MOI of 0.01), and viral titers in the cell culture supernatants at 24 hpi or **(D)** at 48 hpi were determined. **(C)** The percentage reduction of virus titers was detected in the crRNA groups (#1, #2, #3, and #4) relative to the crRNA control group at 24 hpi or **(E)** at 48 hpi. **(F)** HEK-293T cells were cotransfected with Cas13d and the indicated crRNAs plasmid or crRNAs pool. Twenty-four hours later, the cells were infected with SVV (at an MOI of 1), and viral titers in the cell culture supernatants at 48 hpi were determined. **(G)** The percentage reduction of virus titers was detected in the crRNA groups (#1, #2, #3, #4, and mixed pool) relative to the crRNA control group at 48 hpi. Error bars represent the SD; ^*^p < 0.05, ^***^p < 0.001.

To test the antiviral activity of the CRISPR-Cas13d system, we cotransfected CasRx-del-NSL with plasmid containing targeted crRNA or control crRNA in HEK293T cells. Twelve hours later, the transfected cells were infected with SVV at an MOI of 0.01. As expected, we found that in the targeted crRNA group, there was extremely low expression of VP2 (which reflects the replication of SVV) in CasRx-del-NSL-expressing cells (Figure 3B); however, in the control crRNA group, VP2 and CasRx-del-NSL could be easily detected in the same cells (Figure 3A). The colocalization analysis was shown in Figure 3C. This indicated that the CRISPR/Cas13d system could efficiently inhibit SVV replication. To test the anti-SVV activity of four indicated crRNAs, we first evaluated the cell viability of indicated crRNAs transfected HEK293T cells, the result indicated that these crRNAs had not influence on cell viability at 24 and 48 h post-infection (Figure 4A). Next, we quantified the viral titers of the four indicated crRNAs and found that all of the screened crRNAs significantly inhibited SVV replication at 24 and 48 h post-infection (Figures 4B,D). crRNA#2 was the most effective crRNA among the four selected crRNAs. The viral titers of the crRNA#2 group were significantly decreased (by 84%) compared with those of the crRNA control group (Figures 4B,C). In addition, the titers decreased by 74% and 66% in the crRNA#1 and crRNA#4 groups, respectively, compared with those in the crRNA control group (Figure 4C). All targeted crRNA groups showed a relative inhibition ratio of at least 57%. At 48 hpi, the crRNA#2 group maintained a robust ability to inhibit SVV replication, with a decrease of approximately 57% compared to the control group (Figures 4D,E). Furthermore, when compared with control crRNA, crRNA#3, crRNA#1, and crRNA#4 reduced viral titers (by approximately 52%, 43%, and 40%, respectively; Figures 4D,E). Next, we further evaluated the anti-SVV activity at a high MOI (MOI = 1), we get a similar results as MOI = 0.01 (Figures 4F,G). We also evaluated whether these crRNAs had synergetic effect, to our surprised, we failed to observed synergetic effect as CRISPR-Cas9 system (Figures 4F,G; Tang et al., 2017). This needed to be explored in future. Overall, our results indicated that the crRNA consistently mediated robust Cas13d activity in infected cells. In fact, we also evaluate anti-SVV activity of CRISPR-Cas13d in porcine cells, however, all our tested porcine cells manifested a low transfection efficiency (<30%). Therefore, we failed to perform this experiment in porcine cells, we speculated that lentiviral vectors will be an efficient transduction tool to test the anti-SVV activity of CRISPR-Cas13d in future. Furthermore, there are several issues should be concerned in future application, such as whether this CRISPR system has possible off-target effect and how to deliver it efficiently *in vivo*.

In summary, we demonstrated that the CRISPR-Cas13d system was a powerful tool to inhibit SVV replication. In the future, viral vectors or other potential CRISPR-Cas13d carriers may be used for SVV prevention in the clinic.

## Data Availability Statement

The raw data supporting the conclusions of this article will be made available by the authors, without undue reservation.

## Author Contributions

Y-DT, FM, and X-HC designed the experiments. Y-YZ and the other authors performed the experiments. Y-YZ, M-XS, YL, T-YW, M-YJ, CL, MC, Y-ZB, FM, X-HC, and Y-DT analyzed the data. Y-DT and Y-YZ wrote the manuscript. All authors contributed to the article and approved the submitted version.

## Funding

This work was supported by grant from the National Natural Science Foundation of China to FM (32002249) and the State Key Laboratory of Veterinary Biotechnology of CAAS (SKLVBP201803 to Y-DT).

## Conflict of Interest

The authors declare that the research was conducted in the absence of any commercial or financial relationships that could be construed as a potential conflict of interest.

## Publisher’s Note

All claims expressed in this article are solely those of the authors and do not necessarily represent those of their affiliated organizations, or those of the publisher, the editors and the reviewers. Any product that may be evaluated in this article, or claim that may be made by its manufacturer, is not guaranteed or endorsed by the publisher.
